# Disentangling the nuances of diversity ideologies

**DOI:** 10.3389/fpsyg.2023.1293622

**Published:** 2024-01-05

**Authors:** Nicole Russell Pascual, Teri A. Kirby, Christopher T. Begeny

**Affiliations:** ^1^Department of Psychology, University of Exeter, Exeter, United Kingdom; ^2^Department of Psychology, Purdue University, West Lafayette, IN, United States

**Keywords:** diversity, diversity rationales, race, diversity ideology, multiculturalism, colorblindness

## Abstract

**Objectives:**

Minoritized racial groups typically report greater psychological engagement and safety in contexts that endorse multiculturalism rather than colorblindness. However, organizational statements often contain multiple (sub)components of these ideologies. This research broadens our understanding of diversity ideologies in the real-world by: (1) mapping out the content of real-world organizational diversity ideologies, (2) identifying how different components tend to cluster in real-world statements, and (3) presenting these statements to minoritized group members (Study 2) to test how these individual components and clusters are perceived (e.g., company interest, value fit).

**Methods:**

100 US university statements and 248 Fortune 500 company statements were content coded, and 237 racially minoritized participants (*M_age_* = 28.1; 51.5% female; 48.5% male) rated their psychological perceptions of the Fortune 500 statements.

**Results:**

While universities most commonly frame diversity ideologies in terms of value-in-equality, companies focus more on value-in-individual differences. Diversity rationales also differ between organizations, with universities focusing on the moral and business cases almost equally, but companies focusing on the business case substantially more. Results also offered preliminary evidence that minoritized racial group members reported a greater sense of their values fitting those of the organization when considering organizations that valued individual and group differences.

**Conclusion:**

These are some of the first studies to provide a nuanced examination of the components and clusters of diversity ideologies that real-world organizations are using, ultimately with implications for how we move forward in studying diversity ideologies (to better reflect reality) and redesigning them to encourage more diverse and inclusive organizations.

## Introduction

Racial diversity in the United States (US) has increased more quickly than previously predicted (US Census, 2019; Frey, 2020). As racial/ethnic demographics shift in US society, as well as within the workplace, it is crucial to understand how people’s beliefs about how to approach diversity and difference, or their lay *diversity ideologies* (Rattan and Ambady, 2013), impact minoritized racial groups.

Indeed, people hold a range of beliefs about how to approach diversity and difference, and these ideologies can permeate organizational culture (Plaut et al., 2009). Two of the most dominant ideologies primarily differ in whether they highlight group differences (i.e., *multiculturalism*) or downplay them (i.e., *colorblindnesss*; Gündemir et al., 2019). A great deal of scholarship suggests benefits to *highlighting* as opposed to *downplaying* group differences (e.g., Wolsko et al., 2000; Plaut et al., 2009). However, diversity ideologies are far more nuanced than is captured by this broad distinction between multiculturalism and colorblindess. Embedded within each of these broad ideologies, there can be differing messages about *how* exactly to promote diversity (reflecting different diversity ideology components) and *why* (reflecting different diversity cases, or diversity rationales; for an overview, see Gündemir et al., 2019). Thus, the multicultural versus colorblind distinction does not itself allow us to fully understand which components drive marginalized individuals’ reactions to an organization’s ideology, nor the potential beneficial effects of multiculturalism in particular.

In the present research, we document the prevalence of specific components of diversity ideologies and diversity rationales in the real-world to understand the extent to which theoretical understandings of diversity ideologies reflect real-world expressions of ideology (or not). We do so in part by integrating past insights from multiple streams of diversity research, including research on differing diversity ideology components. For example, Gündemir et al. (2017a) distinguish between an emphasis on value-in-group differences, value-in-individual differences, or value-in-similarities (as potentially distinct and defining features of an ideology). Purdie-Vaughns and Walton (2011) further suggest that ideologies might incorporate multiple components, including both value-in-group differences (between-group variability) and value-in-individual differences (within-group variability; arguably rendering a distinct diversity approach/ideology unto itself).

Moreover, we integrate insights on different diversity rationales, including both the ‘moral case’ and ‘business case’ for promoting diversity (Thomas and Ely, 1996). In so doing, we aim to help conceptually bridge these differing streams, offer new insights on how they come together in real organizational settings (100 US universities, 250 companies in the Fortune 500), and more generally highlight the need for more thorough engagement with the nuance and complexity of diversity ideologies – not least to ensure that our understanding of these ideologies does not move forward in a way that is detached from their existence in the real-world.

To ensure our research addressed this gap, we identified components for both how we should navigate diversity (diversity ideology components) and why (diversity rationales) in current research’s diversity ideologies (identified through a literature search conducted by two members of the research team). The key diversity ideologies are summarized in Table 1 (see Supplementary materials for the full analysis). In every paper reviewed, the ideologies used did not reflect a single component in isolation, but a combination. Therefore, our research will code for both the prevalence of the components but also how they group together in real-world diversity statements in both US universities and companies. To help facilitate a more nuanced understanding of why these ideologies are beneficial and for which outcomes, we will also provide an initial, systematic examination of how minoritized racial group members respond when presented with these real-world components (e.g., level of interest in the organization, sense of value fit, authenticity).

**Table 1:** Examples of diversity statement components.

	**Key citation**	**Definition**	**Example of this component**
**Multiculturalism**			
Value-in-group differences	Kirby and Kaiser (2021)	Valuing differences between marginalized social groups	“While other consulting firms mistakenly focus on their staff’s similarities, we train our ethnically diverse workforce to embrace their differences. Focusing on our differences creates a more exciting and collaborative work environment.”
Value-in-diversity	Gündemir et al. (2017b)	Celebrates people of different marginalized racial groups	“Our employees benefit from our dedication to this diversity-focused policy: their own diverse backgrounds are recognized and celebrated through our many diversity initiatives and programs.”
**Colorblindness**			
Value-in-individual differences^1^	Gündemir et al. (2017a)	Focuses on differences at the individual level, such as qualities, experiences or skills	“focusing on individual characteristics creates an exciting work environment”
Value-in-similarities	Purdie-Vaughns et al. (2008)	Focuses on similarities between people	“While other consulting firms mistakenly focus on their staff’s diversity, we train our diverse workforce to embrace their similarities. We feel that focusing on similarities creates a more unified, exciting, and collaborative work environment.”
Value-in-equality	Apfelbaum et al. (2016)	Focuses on equality or prevention of discrimination	“All employees, regardless of background, are treated equally and fairly. Equal opportunity further ensures that our employees are recruited, hired, and promoted without regard to race, sex, age, gender, gender identity or expression, religion, national origin, disability, marital status, sexual orientation, veteran status, or other. “

^1^Value-in-individual differences has also been included under the multicultural categorization (e.g., Gündemir et al., 2017b); “We foster an inclusive and open-minded workplace that values diverse backgrounds and experiences.”

Although colorblindness was once the prevalent ideology in organizations (Plaut, 2002), multiculturalism has seen a dramatic increase (Apfelbaum et al., 2016). This shift in ideology aligns with theoretical and experimental discussions supporting multiculturalism as an identity safety cue for minoritized groups (Gündemir et al., 2019). Two key theories in the field highlight the benefits of valuing differences (multiculturalism ideologies) over similarities (colorblindness) for minoritized groups: acculturation and social identity theories.

Acculturation theories propose that contact between members of different cultural groups results in changes in both groups (Redfield et al., 1936; Graves, 1967). However, minoritized racial groups are particularly responsible for adapting to the majority group and sometimes even suppressing their sub-group identities (Berry, 2001). As cultural and racial identities are a key part of how people perceive themselves (Tajfel and Turner, 1979), particularly for minoritized group members (Gerard and Hoyt, 1974), downplaying those identities—as prescribed by colorblindness—can be detrimental for their self-concept.

Multiculturalism is an ideology that enables minoritized racial groups to preserve their cultural identity (Berry and Kalin, 1995). For minoritized groups, multiculturalism can increase group identification and therefore results in more positive ingroup evaluations (Verkuyten, 2005). Accordingly, compared to colorblindness, minoritized racial groups tend to prefer multiculturalism (Richeson and Nussbaum, 2004). However, there are some important caveats and complexity to this general pattern of findings. When minoritized racial groups are underrepresented in an organization, multiculturalism (versus colorblindness) increases workplace trust, comfort and engagement for minoritized racial groups (Purdie-Vaughns et al., 2008; Plaut et al., 2009), but hurts their performance, persistence, and representation under some circumstances (Apfelbaum et al., 2016). Taken at face value, this might suggest that multiculturalism and colorblindness show discrepant findings for different types of outcomes (e.g., behavioral versus psychological outcomes).

A key driver of these discrepant findings, however, may be the ways researchers frame the ideologies. For example, some research has focused on valuing demographic group differences in their multicultural ideologies (Kirby and Kaiser, 2021), but others have focused on individual (trait) differences (Apfelbaum et al., 2016). Similarly, colorblindness has been defined as a focus on *common ingroup identity* (Dovidio et al., 2007), *valuing equality* (Apfelbaum et al., 2016), *devaluing group identities* (Purdie-Vaughns et al., 2008), *assimilation* (adopting the majority groups’ norms and views; Plaut et al., 2009), and *value-in-individual differences* (i.e., celebrating uniqueness across individuals; Gündemir et al., 2017a).

It is perhaps unsurprising that multiculturalism would create more identity safety for minoritized group members when compared to assimilation or group devaluation (see Hahn et al., 2015). However, even more positive components of colorblindness that focus on *value-in-similarities* suggest it can be detrimental for outcomes such as workplace engagement (Purdie-Vaughns et al., 2008; Plaut et al., 2009). Therefore, it appears that valuing equality (as was the case in Apfelbaum et al.’s (2016) research) is the only exception to the general pattern of colorblindness being detrimental for minoritized racial groups. This aligns with cultural norms, as equality is widely valued in the US (Hofstede, 1980; Thomas and Ely, 1996). Martin Luther King Jr.’s infamous speech captured this by stating that he wished for a world in which we would judge individuals “not by the color of their skin but by the content of their character.” (King, 1963).

In terms of multiculturalism, some research focuses on group differences (Kirby and Kaiser, 2021) and some focuses on value-in-diversity (Verkuyten, 2005; Purdie-Vaughns et al., 2008). As these differing definitions of multiculturalism are being compared to differing definitions of colorblindness, it is difficult to draw meaningful conclusions. For example, focusing on value-in-group differences, compared to value-in-similarities, decreased authenticity and increased perceptions of tokenism for Black Americans who were weakly identified with their racial group (Kirby and Kaiser, 2021). However, *both* value-in-group differences and value-in-individual differences increased minoritized groups’ leadership self-efficacy when compared to value-in-similarities (Gündemir et al., 2017a).

The distinction between focusing on group differences and value-in-diversity is also key. Purdie-Vaughns et al. (2008) found that a multiculturalism ideology, focused on value-in-diversity, increased minoritized groups’ workplace trust and comfort more than a colorblind ideology that devalued group identities. The difference in the valence of these multiculturalism (celebrating) versus colorblindness (devaluing) framings could explain the discrepancy between this finding and some of those discussed previously (see Hahn et al., 2015). Within multiculturalism, there is also a difference in valence between focusing on how groups differ and going one step further to celebrating this diversity (see Table 1 for examples). Our research aims to explore this difference further through documenting the prevalence of both components and their relationships with minoritized groups’ perceptions.

Across the literature, these differences in components of ideologies that are classified under the same or similar terms to multiculturalism versus colorblindness has made it difficult to understand which components drive different findings. These different components of the ideologies represent at least five distinct ideas about navigating diversity (*value-in-group differences, value-in-individual differences, value-in-similarities, value-in-equality,* and *value-in-diversity*). This is the first paper to systematically compare these components, document their presence in the real-world, and provide a better understanding of their effects for the minoritized racial groups they intend to benefit. ^1^

Not only does diversity rhetoric differ in prescriptions for *how* to navigate diversity, but they differ in notions of *why* diversity is important—often called a “case for diversity” or diversity rationale. The two main diversity rationales are the business and moral case. The business case argues that diversity brings economic or instrumental value to the organization through increased productivity, whereas the *moral case* argues that promoting diversity is the right thing to do (Noon, 2007). The *business case* has a number of downsides: (a) it is generally less beneficial for minority groups as it can lead to deprioritization of minority group job applicants, (b) relates to increased graduation rate disparities between White and Black students (Starck et al., 2021), (c) it can lead to concerns from minoritized groups about how they will be treated at work (Ely and Thomas, 2001), (d) it reduces minoritized groups’ sense of belonging (Georgeac and Rattan, 2023), and (e) it makes companies less appealing as employers (Jansen et al., 2021). Despite these downsides, the business case has often been used as the rationale behind multiculturalism (Plaut, 2002). It has even been used as an argument against colorblindness – diversity can be instrumental for the organization (van Knippenberg et al., 2004) and thus the differences that come with diversity should be emphasized rather than downplayed (Gündemir et al., 2019). However, as discussed above, multiculturalism tends to be preferred by minoritized racial groups and therefore, it remains unclear whether the downsides of the business case are also seen in the real-world when coupled with multiculturalism. In practice, multicultural statements can and do make either the business or moral case (or both) and these differences may also contribute to a lack of clarity about why and when multiculturalism versus colorblindness provide identity safety.

This research aims to better understand how the different components of diversity ideologies and rationales are perceived by minoritized racial groups. We will document the components present in both university (Study 1a) and company (Study 1b) diversity statements to understand how their rhetoric might differ. In addition to examining the prevalence of individual components, we will also document which components tend to appear together and whether particular combinations are especially beneficial. Diversity ideologies and rationales have often been studied in isolation and our research aims to understand how these two forms of diversity rhetoric appear together in the real-world.

In addition, we will examine how these components and their clusters relate to psychological measures (Study 2). Specifically, we will investigate the relationships between the company diversity statement components collected in Study 1b and minoritized racial groups’ interest in the company, perceptions of value fit, authenticity, and tokenism.

## Study 1

In Study 1, we assessed the prevalence of different components in real-world university (Study 1a) and company (Study 1b) diversity statements. Specifically, we examined how organizations approach diversity (value-in-group differences, value-in-diversity, value-in-similarities, value-in-individual differences, and value-in-equality) and why diversity matters to them (moral case and business case) in the statements of the top 100 US universities and top 250 Fortune 500 companies. We also assessed what components tend to appear together within the same statements. Because previous research has shown that the private sector focuses more on the business case than the public sector in Dutch organizations (Jansen et al., 2021), we also explored the possibility that there are differences in how Fortune 500 companies (public sector organizations) versus US universities (private sector organizations) discuss diversity (diversity ideologies), and how different diversity ideologies and rationales cluster together.

### Method

#### Study 1a diversity statement coding

We collected diversity statements from the top 100 US universities on the US News and World Report rankings list. Research assistants copied the first block of distinctive text (up until an image or subheading was used) on their diversity and inclusion webpages^2^ and two coders^3^ independently content coded each statement to indicate whether any of the components (*value-in-group differences, value-in-individual differences, value-in-similarities, value-in-equality, value-in-diversity,* the *business case* or the *moral case*) were present (summarized in Table 2; 1 = present, 0 = absent).^4^ Statements could be coded as having multiple components. Once sufficient reliability was achieved (i.e., kappa reliability was at least 0.41, or “moderate” agreement; see Landis and Koch, 1977),^5^ all discrepancies were discussed by the coders to reach a unanimous decision.^6^ These components were our independent variables of interest.

**Table 2:** Content coding of diversity statement components^1^.

Components	Definition	Universities example	Companies example
*Diversity ideology*			
Value-in-group differences	Emphasizes differences between any form of social category (e.g., race, gender, sexual orientation, class, age).	“We recognize and value the unique experiences drawn from differences in race, ethnicity, gender, sexual orientation, age, religion, and veteran status and welcome all students of diverse backgrounds.”	“we define diversity as the range of differences that make individuals unique, including ability, age, ethnicity, gender identification, race, sexual orientation, religious belief and veteran’s status. Inclusion is how we leverage these differences to form a genuine community and expand business opportunities.”
Value-in-individual differences	Emphasizes differences between people or individuals (in a way that is not explicitly about a social group, such as race, gender, sexual orientation, class, age). It focuses on differences in individual qualities and skills.	“By embracing diverse people, ideas, and perspectives we create a vibrant learning and working environment.”	“we take an active, strategic approach to appreciate our individual and collective experiences, different ways of thinking, and various communication styles.”
Value-in-similarities	Emphasizes similarities between people.	“The University of Sterfield is committed to blending our cultures into a harmonious family atmosphere and accepting each as a vital link in our mission.”	“we are united by a culture that cultivates a workplace like no other.”
Value-in-equality	Discusses equality or prevention of discrimination. Equality relates to fairness in terms of equal opportunity for all and ensuring that procedures treat everyone the same way.	“The University of Sterfield is an equal opportunity employer and educator, proudly pluralistic and firmly committed to providing equal opportunity for outstanding men and women of every race, creed and background.”	“We are an equal opportunity employer and strive to build balanced teams from all walks of life.”
Value-in-diversity	Acknowledges or celebrates people of different social groups (e.g., race, gender, sexual orientation, class, age).	“The Council celebrates cultural identities and diversity on campus by fostering awareness and mutual understanding through increased communication.”	“By celebrating diversity across all spectrums, including but not limited to race, national origin, religion, gender, sexual orientation, gender identity, disability, veteran/military status, and age, we are a stronger company and culture.”
*Diversity rationale*			
Business case	Focuses on the benefits diversity brings for the organization itself.	“At The Sterfield University, we recognize that every competitive advantage begins with people. By valuing, celebrating and leveraging the differences and similarities of our students, faculty and staff, we inspire an environment of innovation and passion - one that enables us to create a teaching, research and service environment that better reflects the needs of our students, faculty, staff, customers, constituents, communities and other key stakeholders.”	“We know that diverse teams improve our performance, drive our growth and enhance engagement among ourselves and with our customers and suppliers.”
Moral case	Focuses on valuing diversity and/or equality because it is the right thing to do.	“In an organization so reliant on its people, creating a diverse and inclusive community is not only the right thing to do; it’s critical to the successful implementation of our mission. The greatest challenges facing us in the century ahead are incredibly complex and will require diverse teams who can work collaboratively and innovatively. Actively seeking a student body and a faculty and staff who represent the diversity of our region, nation and world is necessary to prepare our students for an increasingly globalized and connected world.”	“We do the right thing by treating everyone with respect.”

^1^All example statements are anonymized – the company/university name is replaced with “Sterfield.”

#### Study 1b diversity statement coding

We collected diversity statements from the top 250 companies of the Fortune 500 companies. Two of these companies had no diversity statement present, so our final sample size was 248. Four research assistants^7^ followed the same coding procedure as Study 1a (summarized in Table 2).^8^^,^^9^

### Study 1 results

We began by examining the prevalence of diversity ideology components in current universities’ (Study 1a) and companies’ (Study 1b) diversity statements. Next, we examined how these components group together in real-world organizational diversity statements.

#### Prevalence of diversity ideology components

##### Study 1a

In the university statements, value-in-equality (77%) was the most common diversity ideology, followed by value-in-individual differences (69%), value-in-diversity (63%), value-in-group differences (49%), and value-in-similarities (38%). In terms of the ‘why’ of diversity management, the moral case (52%) was more prevalent than the business case (46%), although both appear in nearly half of statements.

##### Study 1b

In the company statements, value-in-individual differences (70.2%) was instead the most common diversity ideology, followed by value-in-equality (53.6%), value-in-diversity (28.6%), value-in-group differences (21.8%), and value-in-similarities (14.5%). Amongst the statements that focus on difference, a focus on value-in-individual differences was more prevalent than value-in-group differences. In terms of the ‘why’ of diversity management, the business case (79.8%) was more prevalent than the moral case (31.9%) – this pattern was similar to university statements, but much more pronounced.

#### How do diversity statement components group together?

We performed a hierarchical agglomerative cluster analysis of the diversity statement ratings to understand how the diversity statement components cluster together.^10^

##### Study 1a

Capturing prominent clusters within university statements, the five-cluster solution is shown in Table 3 and example statements are shown in Table 4. The first cluster – reflecting what we refer to as *Moralistic Value-In-Diversity* – captured 30 statements that were particularly focused on notions of diversity and difference (e.g., *value-in-group differences, value-in-individual differences*, *value-in-diversity*) and *value-in-equality*, framed within a *moral case* for diversity. The second cluster – reflecting *Instrumental Value-In-Diversity* – captured 27 statements that were also focused on notions of diversity and difference (e.g., *value-in-group differences, value-in-individual differences*, *value-in-diversity*) and *value-in-equality*, but were framed within a *business case* for diversity (rather than the *moral case*). Both of these clusters are similar to multicultural meritocracy (Gündemir et al., 2017b; which also focuses on *difference in addition to* value-in-equality), but further distinguishes between the distinct diversity rationales in which they are embedded. The third cluster – reflecting *Instrumental Equality* – captured 20 statements that were high on *value-in-equality, value-in-individual differences,* and the *business case*. The fourth cluster – *Moral Equality* – captured 14 statements that were high on *value-in-equality* and the *moral case*. The fifth cluster – *Dual Identity* – see Gaertner and Dovidio (2000) captured 9 statements high on *value-in-individual differences, value-in-similarities*, and *value-in-equality*, grounded in both the *business case* and *moral case* for diversity.

**Table 3:** Percentage of university statements containing each diversity ideology by cluster (Study 1a).

Cluster	Value-in-group differences	Value-in-individual differences	Value-in-similarities	Value-in-equality	Value-in-diversity	Business case	Moral case
Moralistic Value-In-Diversity	**90.00**	**70.00**	26.70	**76.70**	**100.00**	6.70	**93.30**
Instrumental Value-In-Diversity	**77.8**	**88.90**	40.70	**70.40**	**96.30**	**81.50**	0.00
Instrumental Equality	0.00	**50.00**	25.00	**85.00**	25.00	**65.00**	5.00
Moral Equality	0.00	35.70	42.90	**92.90**	0.00	0.00	**100.00**
Dual Identity	11.10	**100.00**	**88.90**	**55.60**	22.20	**100.00**	**100.00**

Bolded percentages reflect those at or above 50% (i.e., the majority of the statements in that cluster contain the components of interest).

**Table 4:** Example statements for each cluster.

Cluster	Universities example	Companies example
Moralistic Value-In-Diversity	“We envision a Sterfield University where people of all identities & experiences are understood, appreciated, and fully included in the community and where equitable treatment and outcomes prevail.”	N/A
Instrumental Value-In-Diversity	“Sterfield University’s founders opened its doors to all students without regard to religion, race, or gender. Building and sustaining a vibrant community of scholars, students, and staff remains essential to our mission of contributing to, and preparing students to thrive in, an increasingly interconnected world. We strive to create environments for learning, working, and living that are enriched by racial, ethnic, and cultural diversity. We seek to cultivate an atmosphere of respect for individual differences in life experience, sexual orientation, and religious belief, and we aspire to be free of intellectual parochialism, barriers to access, and ethnocentrism. Success in a competitive, global milieu depends upon our ongoing commitment to welcome and engage the wisdom, creativity, and aspirations of all peoples. The excellence we seek emerges from the contributions and talents of every member of the Sterfield University community.”	“We believe achieving success begins with people, and we are focused on building a team with a rich diversity of perspectives, experiences and ideas. As one of the nation’s premier energy companies, Sterfield is committed to recruiting, developing and retaining great people at all levels. A key part of that commitment is to attract and maintain a diverse and multi-generational workforce that can help us meet the continually evolving needs of our customers. To reinforce our commitment, we continue to develop and implement corporate-wide diversity and inclusion training for all of our employees and further strengthen our Corporate Diversity Council and Employee Resource Groups. At Sterfield, we define diversity broadly. We provide an inclusive work environment that is free from discrimination and harassment on the basis of race, color, age, sex, national origin, religion, marital status, sexual orientation, gender identity, gender expression, genetics, disability or protected veteran status. We also appreciate diversity of thought, style, technical and functional capabilities or leadership. When talented employees from varied backgrounds are engaged and contributing to our business success, we all benefit.”
Instrumental Equality	“Everything about our academic mission—teaching, learning, scholarship, research, engagement, and creative activity—is made better by the exchange of ideas and diverse experiences and perspectives of our students, faculty, and staff. We value the contributions and inherent worth of all individuals, and treat others with mutual respect and understanding. And when we are in the field as professionals, we are devoted to understanding the varied historical and social contexts where we work.”	N/A
Moral Equality	“The primary objectives of the programs and services for underrepresented and minority students at Sterfield are to support the outreach, recruitment, and retention of Native American, African American, Hispanic American and those of Pacific Islander heritage. These objectives support the overall campus goal of building a safe, supportive and inclusive community for all students.”	N/A
Dual Identity	“The Office of Institutional Diversity seeks to ensure a University of Sterfield where people of many different backgrounds and perspectives join together to actively advance knowledge. As a community dedicated to scholarship, research, instruction, and public service and outreach, we recognize the importance of respecting, valuing and learning from each other’s differences while seeking common goals.”	N/A
Instrumental Individualism	N/A	“When you bring a variety of perspectives to the table, it creates a culture of innovation—essential to facing the world’s healthcare challenges. We have been widely regarded as an employer of choice, with numerous local and global awards recognizing our commitment to fostering an extraordinary workplace.”
Moralistic Individualism	N/A	“We celebrate the diversity and uniqueness of each employee and believe that everyone has the right to be treated with fairness, dignity, and respect. Our diversity makes us stronger”

##### Study 1b

Within company statements there were fewer prominent clusters. The three-cluster solution is shown in Tables 4, 5. The first cluster – *Instrumental Individualism* – captured 121 statements (49%) that focused on *value-in-individual differences* and the *business case*. The second cluster – *Moralistic Individualism* – captured 59 statements (24%) that that were particularly focused on *value-in-individual differences*, *value-in-equality* and the *moral case*. The third cluster – *Instrumental Value-In-Diversity* – which also appeared in university statements, captured 68 statements (27%) that were particularly focused on diversity and difference (e.g., *value-in-group differences, value-in-individual differences*, *value-in-diversity*) and *value-in-equality*, framed within the *business case*.

**Table 5:** Percentage of company statements containing each diversity ideology by cluster (Study 1b).

Cluster	Value-in-group differences	Value-in-individual differences	Value-in-similarities	Value-in-equality	Value-in-diversity	Business case	Moral case
Instrumental Individualism	0.00	**69.40**	21.50	48.80	0.80	**100.0**	2.50
Moralistic Individualism	0.00	**52.50**	10.20	**52.50**	5.10	35.60	**91.50**
Instrumental Value-In-Diversity	**79.40**	**86.80**	5.90	**63.20**	**98.50**	**82.40**	32.40

Bolded percentages reflect those at or above 50% (i.e., the majority of the statements in that cluster contain the components of interest).

### Study 1 discussion

Although both universities and organizations focus on value-in-similarities the least, universities most commonly advocate for navigating diversity by focusing on value-in-equality, whereas companies focus on value-in-individual differences. The reasons for why diversity should be valued also differ between the organizations, with universities focusing on the moral case and business case almost equally, but companies focusing on the business case substantially more. This is in line with previous work that has shown that the business case is more prevalent in the private sector (Jansen et al., 2021; Georgeac and Rattan, 2023)—this may be because of differences in goals across sectors, among other potential differences. Organizations may implement diversity ideologies to communicate to potential stakeholders that the organization is committed to diversity (i.e., a signaling rationale; Dover et al., 2020). These stakeholders may differ between companies and universities (e.g., potential employees versus students) and therefore so will the nature of the signaling rationale.

The ways statements grouped together also revealed differences between types of organizations. In universities, statements that focus on diversity and difference commonly cluster with either moral reasons for caring about diversity (Moralistic Value-In-Diversity) or business case justifications (Instrumental Value-In-Diversity). However, in companies, only the instrumental Value-In-Diversity statements are seen. The university statements also showed a quadrant with statements either being very high (>75%) on value-in-equality (Instrumental Equality and Moral Equality) or value-in-group differences (Moralistic Value-In-Diversity and Instrumental Value-In-Diversity), and either high on the moral case (Moralistic Value-In-Diversity and Moral Equality) or the business case (Instrumental Value-In-Diversity and Instrumental Equality). For companies, we also found that statements were either high on the moral case (Moralistic Individualism) or the business case (Instrumental Individualism and Instrumental Value-In-Equality). However, we did not find the value-in-equality versus value-in-group differences pattern we found for universities, perhaps as a result of the low prevalence of value-in-group differences.

Moreover, whilst both focus on value-in-individual differences, in universities it tends to come alongside value-in-equality, whereas in companies it is often paired with the business case. Additionally, in universities but not in companies, we found that there is also a grouping that focuses on dual identities (high in value-in-individual differences and value-in-similarities) – this type of ideology recognizes that people belong to individual subgroups whilst also having a shared overarching identity (Glasford and Dovidio, 2011). Overall, these findings suggest much stronger reluctance to focus on group differences in companies as compared to universities and more of a tendency to focus on individualism.

In Study 2, we followed up on these clusters to assess how they are perceived by minoritized racial groups, as well as which individual components drive effects. This allowed us to better determine how rhetoric existing in real organizations impacts on underrepresented groups.

## Study 2

Despite numerous studies examining perceptions of multicultural and colorblind ideologies, it remains unclear which components drive these effects. For example, *why* do minoritized racial groups typically support multicultural over colorblind ideologies (Ryan et al., 2007)? We aimed to address this gap by measuring minoritized racial groups’ responses to the different components discussed thus far, as well as the clusters identified in the organizational statements. To better understand minoritized racial groups’ perceptions of the different components, we assessed their perceptions of 248 Fortune 500 statements on a range of different measures used in previous research in the field^11^^,^^12^^,^^13^. Because of inconsistent operationalizations of diversity ideologies in the literature, we did not initially have strong hypotheses. However, we did expect that the multicultural components (value-in-group differences, value-in-diversity), value-in-equality and the moral case would be associated with more value fit, interest, and authenticity. We expected that value-in-similarities would be negatively associated with these psychological outcomes.^14^

We focused on these dependent measures because previous research has found effects of diversity rhetoric on authenticity (Kirby and Kaiser, 2021), organizational interest (Kirby et al., 2023), and value fit (Purdie-Vaughns et al., 2008). Both lack of value fit and inauthenticity play a key role in reinforcing stereotypes and in turn social inequalities (Schmader and Sedikides, 2018). Therefore, it is key to understand how different concepts of diversity ideologies affect these variables. Moreover, diversity ideologies are often implemented with the intent to appeal to minoritized groups and encourage them to apply (Dover et al., 2020) and therefore it is key to ensure they have this intended effect. We measured tokenism because previous research found that value-in-group differences may increase tokenism (as measured by their prototypicality pressure scale; Kirby and Kaiser, 2021). This finding appears to contradict the general consensus that multiculturalism is universally beneficial (e.g., for value fit; Purdie-Vaughns et al., 2008) for minoritized racial groups (Gündemir et al., 2019), so we wanted to better understand if the different framings in the literature might explain conflicting findings like this one. However, this was an exploratory question because the research suggests that tokenism may be more relevant when accounting for individual differences in identification (Kirby and Kaiser, 2021), which was not possible with the present data

### Method

#### Participants

We recruited racially minoritized participants residing in the US via Prolific. Of the original sample of 269 participants, 32 were excluded as they did not identify as a racial/ethnic minority group member. Therefore, the final sample was 237 participants (28.7% Hispanic or Latino/a, 24.5% Black/African American, 19.4% mixed race other, 12.2% East Asian, 7.6% mixed race Black/White, 7.2% South Asian, 0.4% American Indian/Alaskan Native). Participants were aged between 18 to 69 years old (*M* = 28.13; *SD* = 9.67); 51.5% were female and 48.5% were male, 93.2% were native English speakers.^15^

#### Materials and procedure

This research was approved by the ethics department at the university of the first author, and all participants provided informed consent. Each participant read 10 randomly selected diversity statements from the total pool of 248 statements. The names of the organizations were removed from all statements and replaced with Sterfield—a fictitious name—to prevent prior impressions of the companies affecting the results. Each statement was rated between 6 and 11 times (*M* = 9.56). In analyses, the company interest, value fit, authenticity, and tokenism measures were collapsed for each statement, so that each statement had a single index of average company interest, value fit, authenticity, and tokenism. For all measures, participants responded on a 7-point Likert scale (1 = *Strongly Disagree*, 7 = *Strongly Agree*).

##### Company interest

Participants responded to three items from Kirby et al. (2023): I would be interested in this company; This company would not be a good fit for me (reverse-scored); I would like to work here. Because reliability was low (α = 0.66), we computed company interest as the average of two items (I would be interested in this company; I would like to work here; *r*_SB_ = 0.96). Higher values indicated stronger company interest.

##### Value fit

Participants responded to four items adapted from Purdie-Vaughns et al.’s (2008) trust and comfort scale^16^: I think I would like to work under the supervision of people with similar values as this company; I think I would be treated fairly by my supervisor; I think I would trust the management to treat me fairly; I think that my values and the values of this company are very similar. We computed an average where higher values indicated stronger value fit. Reliability of the measure was excellent (α = 0.97).

##### Authenticity

Participants responded to two items adapted from Kirby and Kaiser’s (2021) authenticity scale^17^: I could be my true self at this company; I would feel comfortable at this company. We computed an average where higher values indicated stronger authenticity (*r*_SB_ = 0.93).

##### Tokenism

Participants responded to five items adapted from Apfelbaum et al.’s (2016) representation-based concerns scale^18^: My performance at this company will only reflect on me, not other racial minorities (R); At this company, I will feel like I have to represent all racial minorities; At this company, I would be concerned that people will treat me differently because of my race; If I don’t do well at this company, it will be viewed as stereotypic of my race; At this company, I do not want to stand out as a racial minority. We computed an average where higher values indicated stronger tokenism. Reliability of the measure was excellent (*α* = 0.79).^19^

Finally, demographic details were collected, and participants were thanked, debriefed, and reimbursed.

Research materials, pre-registration (uploaded before data analysis and an analysis plan is included) and data files are available on OSF: https://osf.io/vfdpc/.

### Results

#### Analytic strategy

Participants were randomly assigned to read 10 diversity statements from the total 248. Rather than using participants as the level of analysis, we used the statements. To do this, we calculated mean company interest, value fit, tokenism, and authenticity ratings for each organization. Our dataset included a row for each company, with the coding from Study 1b and the mean ratings of each dependent variable as separate columns.

We examined whether any clusters of components are especially beneficial (or detrimental). To do this, we used the clusters obtained in Study 1b as an independent variable in ANCOVAs, controlling for word count, on the outcome variables (company interest, value fit, tokenism, and authenticity). Then, we investigated whether any individual components were related to the outcome variables. To investigate this, we ran bivariate correlation analyses between the components and the outcome variables, followed by multiple regression analyses to investigate the relationships between the components and the outcome variables when controlling for the other components and word count.

#### Are particular ideology clusters preferred?

The different clusters were significantly associated with perceptions of value fit and company interest, [*F*(2,242) = 4.06, *p* = 0.018, η_p_^2^ = 0.03] and [*F*(2,242) = 3.58, *p* = 0.029, η_p_^2^ = 0.03], respectively. However, the clusters did not relate to authenticity [*F*(2,242) = 1.91, *p* = 0.150, η_p_^2^ = 0.02] or tokenism [*F*(2,242) = 1.14, *p* = 0.323, η_p_^2^ = 0.01]. Participants reported greater perceptions of value fit and greater company interest for the Instrumental Value-In-Diversity cluster than the Instrumental Individualism and Moralistic Individualism clusters (see Supplementary Table S10). This tentatively provides support for the notion that value-in-diversity and difference fosters fit better than focusing on value-in-individual differences.

#### Which individual diversity ideologies are beneficial?

##### Preliminary analyses

We checked for any multicollinearity issues by running crosstabulation analyses between all of our independent variables (Table 6). Value-in-group differences and value-in-diversity were strongly associated, φ (1, *N* = 248) = 0.81, *p* < 0.001, with only an 8% difference between the scores given to them. Due to multicollinearity concerns (Alin, 2010), these two variables were analyzed separately in two multiple linear regression models. The moral case and business case were also strongly associated, φ (1, *N* = 248) = −0.58, *p* < 0.001, with only a 17% overlap between the scores given to them. To avoid issues with multicollinearity, we deviate from our pre-registered analysis plan by including the moral case and business case in separate models. Below we report the findings from the regression models including the moral case and value-in-group differences. The Supplementary materials include the models with value-in-diversity and the business case.

**Table 6:** Cramer’s phi values for associations between independent variables.

	1	2	3	4	5	6	7
1. Value-in-group differences	–						
2. Value-in-individual differences	0.22***	–					
3. Value-in-similarities	−0.13*	−0.06	–				
4. Value-in-equality	0.01	0.14*	−0.03	–			
5. Value-in-diversity	0.81***	0.18**	−0.11	0.09	–		
6. Business case	0.05	0.20**	0.04	<0.01	−0.02	–	
7. Moral case	−0.05	−0.12	−0.04	0.15*	0.03	−0.58***	–

*p ≤ 0.05, **p ≤ 0.01, ***p ≤ 0.001.

##### Company interest

Correlation analyses revealed that minoritized racial groups were more interested in working for companies with value-in-group differences, value-in-individual differences, value-in-equality, value-in-diversity, and the business case in their statements (Table 7). The regression analyses showed that only the value-in-individual differences effect held when controlling for the other components (Table 8 and Supplementary Tables S11–S13).

**Table 7:** Correlations between diversity ideology components and outcome variables.

	*M*	*SD*	Value fit	Authenticity	Tokenism	Value-in-group differences	Value-in-individual differences	Value-in-similarities	Value-in-equality	Value-in-diversity	Business case	Moral case
Company interest	5.19	0.55	0.93***	0.90***	−0.63***	0.22***	0.25***	−0.04	0.24***	0.26***	0.14*	0.03
Value fit	5.21	0.51	–			0.25***	0.23***	−0.05	0.23***	0.25***	0.15*	0.03
Authenticity	5.06	0.56	0.91***	–		0.21***	0.18**	−0.07	0.21***	0.23***	0.10	0.05
Tokenism	3.48	0.44	−0.63***	−0.64***	–	−0.15*	−0.13*	0.06	−0.21***	−0.18**	−0.07	−0.08
Word count	100.90	65.98				0.24***	0.20***	−0.05	0.41***	0.37***	0.16*	0.10
*Percentage*						21.8	70.2	14.5	53.6	28.6	79.8	31.9

*p ≤ 0.05, **p ≤ 0.01, ***p ≤ 0.001.

**Table 8:** Relationship between diversity statement components and psychological measures with value-in-group differences and moral case in model.

	**Interest**			**Value fit**			**Authenticity**			**Tokenism**		
	*R^2^* = 0.23, *F*_6,241_ = 11.64, *p* < 0.001			*R^2^* = 0.20, *F*_6,241_ = 10.19, *p* < 0.001			*R^2^* = 0.17, *F*_6,241_ = 8.47, *p* < 0.001			*R^2^* = 0.14, *F*_6,241_ = 6.66, *p* < 0.001		
**Predictor**	**β**	***t***	***p***	**β**	***t***	***p***	**β**	***t***	***p***	**β**	***t***	***p***
Value-in-group differences	0.10	1.65	0.101	0.14	2.26	0.025	0.11	1.79	0.075	−0.05	−0.82	0.412
Value-in-individual differences	0.15	2.49	0.013	0.12	2.04	0.043	0.09	1.40	0.162	−0.06	−0.88	0.381
Value-in-similarities	<0.01	0.01	0.993	−0.01	−0.11	0.912	−0.04	−0.60	0.548	0.03	0.49	0.622
Value-in-equality	0.06	0.98	0.326	0.08	1.19	0.234	0.05	0.77	0.442	−0.06	−0.96	0.336
Moral Case	<0.01	0.08	0.939	0.01	0.08	0.937	0.03	0.46	0.646	−0.05	−0.79	0.433
Word Count	0.35	5.45	<0.001	0.31	4.71	<0.001	0.32	4.73	<0.001	−0.30	−4.47	<0.001

##### Value fit

Correlation analyses revealed that minoritized racial groups had higher value fit perceptions for companies with value-in-group differences, value-in-individual differences, value-in-equality, value-in-diversity, and the business case in their statements (Table 7). The regression analyses revealed that only the value-in-individual differences and value-in-group differences effects held when controlling for the other components (Table 8 and Supplementary Table S11). It is key to note that the findings for the relationship between value-in-individual differences and value fit are not significant in two of the models that account for multicollinearity issues (*p* = 0.072 when value-in-group differences and the business case are included, and *p* = 0.051 when value-in-diversity and the business case are included; see Supplementary Tables S12, S13).

##### Authenticity

Correlation analyses revealed that minoritized racial groups felt like they could be more authentic in companies with value-in-group differences, value-in-individual differences, value-in-equality and value-in-diversity in their statements (Table 7). The regression analyses revealed that none of these effects held when controlling for the other components (Table 9 and Supplementary Tables S11–S13).

**Table 9:** Indirect effects from parallel mediation model in Study 2.

Mediator	*b*	*SE*	95% CI
Value Fit	0.11	0.04	[0.02, 0.19]
Authenticity	0.04	0.02	[<0.01, 0.09]
Tokenism	<0.01	<0.01	[<0.01, 0.01]

##### Tokenism

Correlation analyses revealed that minoritized racial groups felt like they would be tokenized less in companies with value-in-group differences, value-in-individual differences, value-in-equality and value-in-diversity in their statements (Table 7). The regression analyses revealed that none of these effects held when controlling for the other components (Table 8 and Supplementary Tables S11–S13).

##### Mediation tests

We ran a parallel mediation model to investigate whether the relationship between value-in-individual differences and company interest was mediated by value fit, authenticity, and/or tokenism (controlling for word count). We found that only value fit showed a significant indirect effect on interest (see Table 9 for statistics).

### Study 2 discussion

This study aimed to disentangle a range of diversity ideologies and examine how their clusters and individual components relate to psychological measures. Racial minority group members reported greater perceptions of value fit and company interest for the Instrumental Value-In-Diversity cluster than the Instrumental Individualism and Moralistic Individualism clusters. When these clusters were broken down into the individual components of diversity ideologies, value-in-individual differences and value-in-group differences were associated with a stronger sense of value fit. However, only value-in-individual differences related to company interest, which was mediated by value fit. We also found that increases in word count relate to more positive perceptions of the statements, irrespective of content.

Our research is in line with previous research that began to disentangle the different components in the literature. Gündemir et al.’s (2017a) research distinguished between a focus on value-in-individual differences or value-in-group differences and found both related to increased leadership self-efficacy. Similarly, we found that both components relate to increased value fit.

This increase in value fit resulting from value-in-individual differences is associated with an increase in company interest. These findings enable us to better understand conflicting findings in previous literature. It was unclear whether value-in-individual differences was beneficial for minoritized groups. In one instance it was compared to value-in-equality where it was relatively detrimental for their performance when highly underrepresented (Apfelbaum et al., 2016). In other instances, it was compared to value-in-similarities where it improved minoritized groups’ leadership self-efficacy (Gündemir et al., 2017a). When disentangling the components, it continued to signal fit and facilitate organizational interest among minoritized racial groups.

## General discussion

This paper had two key aims. The first was to examine which diversity ideologies are commonly used by organizations. The second was to disentangle a range of diversity ideologies and examine which clusters and components are related to minoritized racial groups’ psychological perceptions.

In terms of the components used by universities and companies, both types of organizations focus on value-in-similarities the least. However, universities most commonly focus on value-in-equality, whereas companies focus more on value-in-individual differences. Value-in-individual differences is also coupled with value-in-equality in universities but not in companies. The reasons for why diversity should matter also differ between them, with universities focusing on both approaches equally and companies focusing on the business case more. In universities, we also found that statements that focus on diversity and differences commonly cluster with either moral reasons for caring about diversity (Moralistic Value-In-Diversity) or the business case (Instrumental Value-In-Diversity). In companies, a focus on diversity and differences commonly appears alongside the business case (Instrumental Value-In-Diversity), but potentially due to the low prevalence of the moral case the Moralistic Value-In-Diversity ideology was not found. Previous research has suggested that different types of organizations differ in their reasons for caring for diversity (Jansen et al., 2021), and our research suggests they also differ in how they navigate diversity.

Most importantly, focusing on both individual and group differences relates to increased value fit. For value-in-individual differences, this increase in value fit also in turn relates to increased company interest. The benefits of focusing on both of these components align with Gündemir et al.’s (2017a) research which found that both components increase minoritized groups’ leadership self-efficacy. This also aligns with the identity safety literature, which has proposed an ideology that goes beyond the focus on group differences (between-group variability) but also acknowledges value-in-individual differences (within-group variability) may foster a sense of belonging amongst minoritized racial groups (Purdie-Vaughns and Walton, 2011).

Value-in-group differences relating to increased value fit also aligns with acculturation and social identity theories. Acculturation theories propose that valuing group differences enables minoritized racial groups to maintain their ethnic identities in cultures where many ethnic groups are present (Berry, 2001). Social identity theory further argues that valuing group differences increases group identification and positive ingroup evaluations among minority groups (Verkuyten, 2005). Our ethnic identities are key to our self-concepts (Tajfel and Turner, 1979). Therefore, it is logical that a diversity ideology that enables minoritized racial groups to preserve and strengthen their social identities would align with their values.

Similarly, the benefits of value-in-individual differences fit within the current cultural context. In the US, individualism is highly valued and on the rise (Twenge et al., 2013), albeit less so for minoritized racial groups (Vargas and Kemmelmeier, 2013). However, these findings appear to conflict with other research, at least on the surface. When organizations define diversity “broadly,” focused on a wide range of individual characteristics, minoritized racial groups report less interest in those organizations (Kirby et al., 2023). However, it only hurts their interest if the organization neglects to explicitly mention minoritized groups. Similarly, our research showed the Instrumental Value-In-Diversity ideology—which combines value-in-group differences with value-in-individual differences was associated with increased value fit and interest relative to ideologies that did not include group differences. Thus, value-in-individual differences has clear benefits for organizational interest, but it may not always be sufficient on its own without acknowledging important social identities. These findings also align with scholarly perspectives suggesting that acknowledging a wide range of disadvantaged groups might harness the benefits of multiculturalism without making individuals feel tokenized (Rios and Cohen, 2023).

These detrimental effects of solely focusing on value-in-individual differences (without value-in-group differences) may also explain why we did not find the moral case was positively related to minoritized groups’ perceptions of the statements as expected. The moral case tends to cluster with individual differences (Moralistic Individualism) and therefore the downsides of only focusing on individual differences may have prevented the benefits of the moral case from being detected. Investigating whether a Moralistic Individualism ideology that also includes the value-in-group differences component is perceived more positively by minoritized racial groups would be an interesting avenue for future research.

We also did not find any effects for our authenticity or tokenism dependent measures. This may be because these effects are moderated by participant level variables. Previous research (Kirby and Kaiser, 2021) found that the relationship between diversity rhetoric and authenticity is moderated by identification. As our data was analyzed at the statement level not the participant level, we were unable to test whether identification moderated our findings. Also, participants were only presented with a company diversity statement compared to previous research which has provided more information on the company context (e.g., Apfelbaum et al., 2016). As authenticity and tokenism are more abstract than company interest and value fit, our methodology may not have sufficed for authenticity and tokenism effects to be detected, as they may require a fuller understanding of the company context.

### Theoretical and practical implications

This research contributes to the field in being the first paper to document the prevalence of diversity ideologies and rationales in real-world diversity statements, as well as how they tend to cluster together. This enabled us to begin to understand how the ideologies and rationales numerous papers have studied are implemented in organizations. For example, researchers tend to define value-in-similarities in terms of similarities between members of the organization (e.g., Purdie-Vaughns et al., 2008), whereas in practice value-in-similarities focused more on having a unified and cohesive culture. Alternatively, this finding could also reflect a shift over time in how organizations frame a focus on similarities.

We also assessed how minoritized racial groups perceive these components and the ways they group together. The Instrumental Value-In-Diversity ideology (value-in-group differences, value-in-individual differences, and value-in-diversity, value-in-equality, and the business case), positively related to minoritized groups’ psychological perceptions. Most organizations adopt a multicultural approach (Apfelbaum et al., 2016) and our research suggests that organizations should frame multiculturalism in terms of the Instrumental Value-In-Diversity ideology. Our individual components analysis suggested the positive effects of the Instrumental Value-In-Diversity ideology were driven by value-in-individual differences and group differences, so implementation of the Value-In-Diversity ideology should ensure these components are prioritized. However, as this study was correlational, it is important for further research using experimental methods to assess if these effects are causal before implementation.

### Constraints on generality

Whilst this research was the first to disentangle the different diversity ideology components, diversity ideologies are only a proxy of what companies’ diversity management is like in practice. Further research should investigate whether diversity ideologies match company diversity practices, as well as how the company’s overall diversity climate relates to minoritized racial groups’ psychological perceptions. We also used a sample from the US, and these results may differ in other countries with different racial relations. They may also differ across different racially minoritized groups, but we did not have sufficient power to be able to differentiate between different groups. As perceptions of discrimination differ between racial groups (Bonilla-Silva and Dietrich, 2011; Keum et al., 2018), it is key for further research to investigate this. We also categorized participants as minoritized racial groups by asking them to self-report their race/ethnicity. Although this is typical in psychological research, future studies could confirm that these participants themselves identify as minoritized. Moreover, due to the complex nuances of the components, inter-rater reliabilities were low for some components. Finally, we have not tested these questions experimentally, which means we cannot make strong claims about causality. However, using a large range of real diversity statements is nonetheless a strength of the research.

## Conclusion

Universities and companies differ in how they frame their diversity policies, with companies focusing most heavily on celebrating value-in-individual differences and universities focusing on value-in-equality. This focus on celebrating difference matches well with the needs of racially minoritized people – expressing a value for individual, as well as group, differences facilitates a stronger sense that a company’s values fit with their own. These findings have important implications for the nuances of how organizations should frame their diversity strategies in order to foster identity safety among minoritized groups.

## Data availability statement

The datasets presented in this study can be found in online repositories. The names of the repository/repositories and accession number(s) can be found at: https://osf.io/vfdpc/.

## Ethics statement

The studies involving humans were approved by the Psychology ethics committee at the University of Exeter. The studies were conducted in accordance with the local legislation and institutional requirements. The participants provided their written informed consent to participate in this study.

## Author contributions

NRP: Conceptualization, Data curation, Formal analysis, Funding acquisition, Investigation, Methodology, Project administration, Resources, Supervision, Validation, Visualization, Writing – original draft, Writing – review & editing. TK: Conceptualization, Formal analysis, Funding acquisition, Investigation, Methodology, Project administration, Resources, Supervision, Validation, Writing – original draft, Writing – review & editing. CB: Visualization, Writing – review & editing.

## Footnotes

^1^Research with White people has shown that when colorblindness is treated as a multifaceted construct rather than unidimensional, the different components are associated with different prejudice outcomes (Whitley et al., 2022).

^2^For organizations where diversity statements appeared in multiple locations, we used their diversity and inclusion page. For organizations that did not have a diversity and inclusion page, we searched the website for other places where the diversity statement could appear (e.g., careers or about us pages) and used those.

^3^The two coders were a White British/Spanish woman and an Asian woman who were long-term residents of the United Kingdom. The coding for the business case and moral case were conducted later and included a White French and a White British woman for the business case and two White women for the moral case.

^4^The subjectivity of the coders may have influenced our results. For example, the interpretation of a White woman may differ from the interpretation of an Asian woman. However, during the first iterations of the coding process, we adapted our coding scheme so that different coders would have similar interpretations (i.e., until we obtained sufficient reliability).

^5^Value-in-group differences: *κ* = 0.66; coder agreement = 83%, Value-in-individual differences: *κ* = 0.54; coder agreement = 81%, Value-in-similarities: *κ* = 0.65; coder agreement = 84%, Value-in-equality: *κ* = 0.60; coder agreement = 87%, Value-in-diversity: *κ* = 0.77; coder agreement = 89%, Business case: *κ* = 0.56; coder agreement = 78%, Moral case: *κ* = 0.52; coder agreement = 76%.

^6^After coding the full set, three categories did not have sufficient reliability. After revising the coding scheme and recoding, value-in-similarities, value-in-equality, and value-in-diversity did not have sufficient reliability, but we attained sufficient reliability after one, one, and two more iterations, respectively. The business and moral cases were coded separately and required one iteration of coding the full set.

^7^The four coders were three White women and one Asian woman. The coding for the business case and moral case were conducted later and included a White French and a White British/Spanish woman.

^8^Sufficient reliability was achieved for all components: Value-in-group differences (*κ* = 0.74; coder agreement = 91%), Value-in-individual differences: (*κ* = 0.50; coder agreement = 80%), Value-in-similarities: (*κ* = 0.51; coder agreement = 88%), Value-in-equality: (*κ* = 0.64; coder agreement = 82%), Value-in-diversity: (*κ* = 0.66; coder agreement = 88%), Business case: (*κ* = 0.53; coder agreement = 81%), Moral case: (*κ* = 0.44; coder agreement = 77%).

^9^For all categories, we attained sufficient reliability in one iteration of coding the full set of statements.

^10^This analysis was performed in SPSS using the squared Euclidean distance similarity measure and the Ward’s method (Ward, 1963). The Ward’s method was selected as it gives more effective solutions than other methods for binary data (Hands and Everitt, 1987; Tamasauskas et al., 2012). The number of clusters was determined through an analysis of the dendrogram and agglomeration schedule following Yim and Ramdeen’s (2015) recommendations. Based on Clatworthy et al.’s (2005), also see Jolliffe et al.’s (1982) recommendation, to assess the validity of the cluster structure, we removed variables and re-ran analyses. This suggested that our clusters were robust.

^11^We also ran a similar preliminary study with the university statements. However, because it only had 100 statements, it was underpowered. For simplicity, we focus on outcomes for the company statements and only report the study with university statements in the online supplement.

^12^ https://osf.io/q5h7f

^13^We amended our original pre-registration before data analysis to clarify that we would only include variables that were significantly associated with our dependent variables in the mediation analyses.

^14^Here, we discuss our original hypotheses, which were somewhat exploratory. However, after some unexpected findings in a preliminary (underpowered) study, we pre-registered more specific hypotheses. These hypotheses were mostly in line with the above predictions, with the exception of predicting that value-in-group-differences would predict increased feelings of tokenism. However, we have de-emphasized this hypothesis for the sake of clarity because we did not replicate the preliminary finding – more details and justification for this decision can be found in the online supplement. Some of the pre-registered analyses are also being included in a separate manuscript focused on real-world diversity outcomes (e.g., workplace inclusion indices and representation of minoritized racial groups), rather than the current focus on perceptions of diversity statements.

^15^SES was not collected due to time and resource constraints.

^16^We excluded any items that measured authenticity or company interest and changed any references to the company they used in their manipulation to ‘this company’.

^17^The four items in this measure were very similar to one another so we selected the two most distinct items in the interest of shortening the questionnaire.

^18^We excluded one item “My [gender/race] would be very important to me at Redstone” that did not capture tokenism – instead identity centrality. We also changed any references to the company they used in their manipulation to ‘this company’.

^19^Tokenism was also measured with a single item “At this company, I would be seen as the same as other members of groups to which I belong” for comparison with the university data that is reported in the supplement.
