# Supplementary material for: Disentangling the nuances of diversity ideologies
Source: Front Psychol. 2024 Jan 5;14:1293622. doi: 10.3389/fpsyg.2023.1293622 (PMC10799561; doi:10.3389/fpsyg.2023.1293622)
Supplement: Supplementary file 1 [file Data_Sheet_1.docx]

**Disentangling the Nuances of Diversity Ideologies**

**Supplementary Materials**

**Contents Page**

Introduction ...............................................................................................................................1

Dendrograms ..............................................................................................................................7

University Study ........................................................................................................................8

Methods ………………………………………………………………………………..8

Results …………………………………………………………………………………9

Discussion ……………………………………………………………………………17

Additional Tables ……………………………………………………………….……18

Study 2 .....................................................................................................................................20

**Introduction**

| **Table S1**  Previous Literature Diversity Statement Components Coding | | | | | | |
| --- | --- | --- | --- | --- | --- | --- |
| **Reference** | Apfelbaum et al. (2016) | | | | Purdie-Vaughns et al. (2008) | |
|  | Items/Coding | | Manipulation | | Manipulation | |
| **Diversity ideology**  **(most beneficial**  **italicised)** | *Value-in-equality* | Value in difference | *Value-in-equality* | Value in difference | Colorblindness | *Valuing diversity* |
| Value-in-individual differences |  |  |  | X |  |  |
| Value-in-group differences |  | X |  |  |  |  |
| Value-in-diversity |  | X |  |  |  | X |
| Minimizing attention to  differences | X |  | X |  | X |  |
| Value-in-similarities |  |  |  |  | X |  |
| Assimilation |  |  |  |  |  |  |
| Equality | X |  | X |  | ? | X |
| Moral Case | X |  |  |  |  |  |
| Business Case |  | X | X | X | X | X |
| Meritocracy |  |  | X |  |  |  |
| Mentions demographics |  |  | X |  | X (devalue it) |  |

| **Reference** | Plaut et al. (2009) | | Gündemir et al. (2017a) | | |
| --- | --- | --- | --- | --- | --- |
| **Diversity ideology**  **(most beneficial**  **italicised)** | Colorblindness | *Multiculturalism* | Value-in-homogeneity | *Multiculturalism* | *Value-in-individual differences* |
| Value-in-individual differences |  |  |  |  | X |
| Value-in-group differences |  | X | X | X |  |
| Value-in-diversity |  | X | ? | X |  |
| Minimizing attention to differences | X |  | X |  | X |
| Value-in-similarities | X |  | X |  | X |
| Assimilation | X |  |  |  |  |
| Equality |  |  |  |  |  |
| Moral Case |  |  |  |  |  |
| Business Case |  |  | X | X | X |
| Meritocracy |  |  |  |  |  |
| Mentions demographics |  |  | X | X |  |

| **Reference** | Gündemir et al. (2017b) | | | Kirby & Kaiser (2021) | |
| --- | --- | --- | --- | --- | --- |
| **Diversity ideology (most beneficial italicised)** | Value-in-merit | Multiculturalism | *Multicultural meritocracy* | *Colorblindness* | Multiculturalism |
| Value-in-individual differences |  | X | X |  |  |
| Value-in-group differences | ? | ? | ? |  | X |
| Value-in-diversity | X | X | X |  | ? |
| Minimizing attention to differences |  |  |  | X |  |
| Value-in-similarities |  |  |  | X |  |
| Assimilation |  |  |  |  |  |
| Equality | X |  | X |  |  |
| Moral Case |  |  |  |  |  |
| Business Case | X | X | X | X | X |
| Meritocracy | X |  | X |  |  |
| Mentions demographics | X | X | X | X (devalue it) | X |

| **Reference** | Wolsko et al. (2000) | | Starck et al. (2021) | | | |
| --- | --- | --- | --- | --- | --- | --- |
|  | Manipulation | | Manipulation | | Coding | |
| **Diversity ideology (most beneficial italicised)** | Colorblindness | *Multiculturalism* | Instrumental | *Moral* | Instrumental | *Moral* |
| Value-in-individual differences | X |  |  |  | X |  |
| Value-in-group differences |  | X | ? |  |  |  |
| Value-in-diversity |  | X | X |  |  | X |
| Minimizing attention to differences | X |  |  |  |  |  |
| Value-in-similarities | X | ? |  |  |  |  |
| Assimilation |  |  |  |  |  |  |
| Equality |  |  |  | X |  | X |
| Moral Case |  |  |  | X |  | X |
| Business Case |  | X | X |  | X |  |
| Meritocracy |  |  |  |  |  |  |
| Mentions demographics |  | X |  |  |  |  |

| **Reference** | Trawalter et al. (2016) | | Jansen et al. (2021) | |
| --- | --- | --- | --- | --- |
| **Diversity ideology (most beneficial italicised)** | Diversity is good | *Diversity is fair* | Business | *Moral* |
| Value-in-individual differences | X |  | X | X |
| Value-in-group differences |  |  |  |  |
| Value-in-diversity |  | X |  |  |
| Minimizing attention to differences |  |  |  |  |
| Value-in-similarities |  |  |  |  |
| Assimilation |  |  |  |  |
| Equality |  | X | X | X |
| Moral Case |  | X |  | X |
| Business Case | X |  | X |  |
| Meritocracy | X |  |  |  |
| Mentions demographics |  |  |  |  |

**Study 1**

**Study 1a Dendrogram**


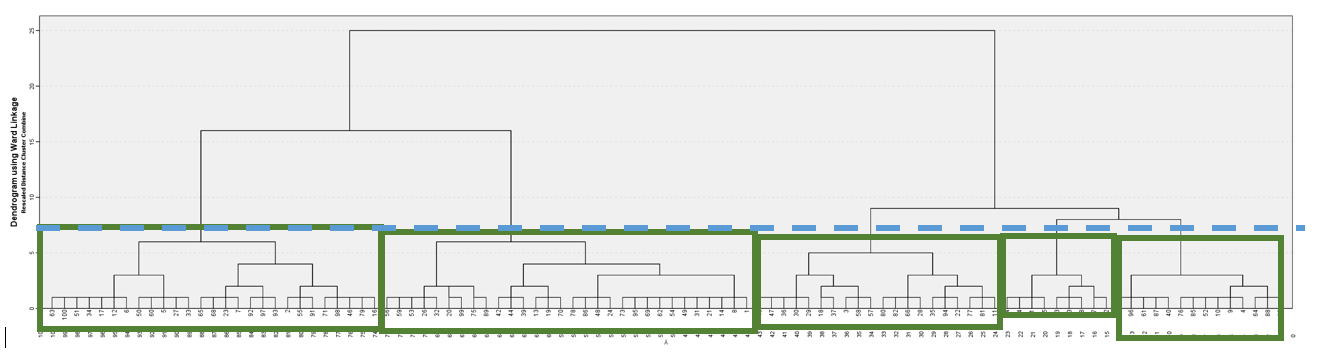


**Study 1b Dendrogram**


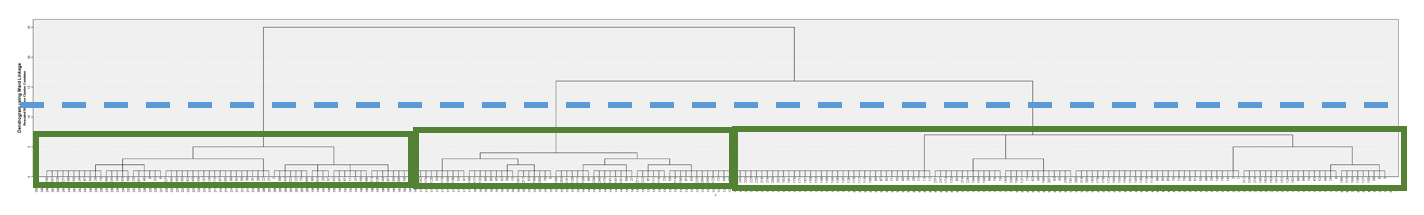


**University Study**

We present this data here rather than in the main text for the purpose of simplicity as this study was underpowered and therefore, we had to interpret the findings cautiously.

**Methods**

**Participants**

One hundred participants from various racial/ethnic minority backgrounds (26% Black/African American, 21% Hispanic or Latino/a, 13% East Asian, 5% American Indian/Alaskan Native, 4% mixed race other, 3% South Asian, 3% another group, 1% mixed race Black/White, 24% did not report) living in the US were recruited via Amazon’s Mechanical Turk. Participants were reimbursed $0.75 to thank them for their participation. Seventy seven participants completed the study and thus completed the demographic details section (33.3% male, 65.3% female, 1.3% other, 1 did not report; they were between 18 to 60 years old (*M* = 30.45; *SD* = 8.40); 96% native English speakers). Partial data was retained to maximise statistical power.

One hundred and seventy White participants living in the US were recruited via Amazon’s Mechanical Turk. Participants were reimbursed $0.75 to thank them for their participation. One hundred and thirty three participants completed the study and thus completed the demographic details section (32.3% male, 67.7% female; they were between 19 to 73 years old (*M* = 38.68; *SD* = 12.52); 98.5% native English speakers). Partial data was retained to maximise statistical power.

**Materials and Procedure**

Participants completed a screening questionnaire where they reported demographic information, including racial background, gender and age. Next, participants read ten randomly selected diversity statements from the pool of statements and completed support and tokenism measures. The names of the universities were removed from all statements and replaced with Sterfield—a fictitious name—to prevent prior impressions of the universities affecting the results. Finally, demographic details were collected, and participants were thanked, debriefed, and reimbursed.

***Ideology Support***

For each statement, participants responded on a 7-point Likert scale (1= *Strongly Disagree*, 7 = *Strongly Agree)* to three items: “I agree with this university statement”; “I like this university statement”; “I support this university statement”. We computed an average where higher values indicated stronger support for the statement. Reliability of the measure was excellent (α = .96 for the minoritized racial groups datafile and α = .98 for the White participants datafile). In analyses, the support measure was collapsed for each statement, so that each statement had a single index of average support. Each statement was rated between 5 and 11 times (*M* = 8.6) by minoritized racial groups and between 9 and 17 times (*M* = 14.0) by White participants.

***Tokenism***

Tokenism was measured with a single item “At this university, I would be seen as the same as other members of groups to which I belong”.

**Results**

***Analytic Strategy***

Because participants were randomly assigned to read 10 diversity statements from the total 100, rather than using participants as the level of analysis, we used the statements. To do this, we calculated mean ideology support and tokenism ratings for each university.

We examined whether any clusters of components affect psychological measures. To do this, we used the clusters obtained in Study 1a in an ANCOVA on the outcome variables, controlling for word count.

Next, we ran bivariate correlations between the components and psychological measures (ideology support and tokenism). Following these analyses, we ran multiple regression analyses to investigate the relationships between the components and the outcome variables when controlling for the other components and word count.

***Are Particular Clusters of Ideologies Preferred?***

One-way ANCOVAs examining the different clusters of ideologies on support, controlling for word count, revealed no differences across clusters, *F*(4,90) = 1.34, *p* = .262, η_p_^2^ = .06.^^[[1]](#footnote-1)^^ Because the study was underpowered (sensitivity analysis indicated the minimum detectable effect size was η_p_^2^ = .11), we nonetheless examined means and post-hoc comparisons (Table S2). Racial minority group members supported the moralistic value-in-diversity cluster significantly more than the instrumental value-in-diversity cluster. One-way ANCOVAs on tokenism controlling for word count revealed that tokenism did not significantly differ across the clusters *F*(4,90) = 1.05, *p* = .388, η_p_^2^ = .04.^^[[2]](#footnote-2)^^ Because the study was underpowered (sensitivity analysis indicated the minimum detectable effect size was η_p_^2^ = .11), we nonetheless examined means and post-hoc comparisons (Table S3) and found no significant differences.

| **Table S2. Post-Hoc Comparisons Using the LSD Test for the Effect of the Clusters on Support** | | | | | | | |
| --- | --- | --- | --- | --- | --- | --- | --- |
|  |  | | *p*-values for LSD Comparisons | | | | |
| Cluster | *M* | *SD* | 1 | 2 | 3 | 4 | 5 |
| Moralistic Value-In-Diversity | 5.51 | .41 | - |  |  |  |  |
| Instrumental Value-In-Diversity | 5.31 | .41 | .035 | - |  |  |  |
| Instrumental Equality | 5.33 | .31 | .360 | .691 | - |  |  |
| Moral Equality | 5.39 | .45 | .352 | .396 | .827 | - |  |
| Dual identity | 5.25 | .63 | .093 | .886 | .652 | .422 | - |

| **Table S3. Post-Hoc Comparisons Using the LSD Test for the Effect of the Clusters on Tokenism** | | | | | | | |
| --- | --- | --- | --- | --- | --- | --- | --- |
|  |  | | *p*-values for LSD Comparisons | | | | |
| Cluster | *M* | *SD* | 1 | 2 | 3 | 4 | 5 |
| Moralistic Value-In-Diversity | 4.92 | .37 | - |  |  |  |  |
| Instrumental Value-In-Diversity | 4.79 | .41 | .379 | - |  |  |  |
| Instrumental Equality | 4.49 | .50 | .116 | .314 | - |  |  |
| Moral Equality | 4.70 | .55 | .139 | .484 | .645 | - |  |
| Dual identity | 4.65 | .46 | .110 | .350 | .862 | .758 | - |

***Relationship Between Diversity Ideology Components with Psychological Measures***

**Preliminary Analyses.** We checked for any multicollinearity issues (Alin, 2010) by running crosstabulation analyses between all of our IVs (Table S4). Value-in-group differences and value-in-diversity were strongly associated, φ (1, N = 100) = .75, *p* < .001, with only a 14% difference between the scores given to them. Similarly, the moral and business cases were strongly associated, φ (1, N = 100) = -.52, *p* < .001, with only a 24% overlap between the scores given to them. Because of the strong overlap, we focused on value-in-group differences and on the moral case in the main analyses, only reporting the models with value-in-diversity and business case in the additional tables section of this document.

| **Table S4. Cramer’s Phi Values for Associations Between Independent Variables** | | | | | | | |
| --- | --- | --- | --- | --- | --- | --- | --- |
|  | 1 | 2 | 3 | 4 | 5 | 6 | 7 |
| 1. Value-in-group differences | - |  |  |  |  |  |  |
| 2. Value-in-individual differences | .27** | - |  |  |  |  |  |
| 3. Value-in-similarities | -.15 | .12 | - |  |  |  |  |
| 4.Value-in-equality | .01 | -.11 | -.11 | - |  |  |  |
| 5. Value-in-diversity | .75*** | .29** | -.17 | -.03 | - |  |  |
| 6. Business case | -.18 | .23* | .23* | -.12 | -.04 | - |  |
| 7. Moral case | .02 | -.08 | .01 | .09 | -.07 | .52*** | - |
| *Note.* * *p* ≤ .05 ** *p* ≤ .01 *** *p* ≤ .001 | | | | | | | |

**Psychological Measures*.*** Correlation analyses revealed that none of the components were associated with support from minoritised racial groups. Unexpectedly, statements that focused on value-in-group differences and value-in-diversity were associated with increased tokenism by racial minority group members (Table S5).^^[[3]](#footnote-3)^^ The regression analyses revealed that only the value-in-group differences tokenism effect held when controlling for the other components (Table S6 and Tables S7-9). However, the R-squared value of the model was low and we used a single item measure for tokenism, so we interpreted this finding cautiously before replication.

| **Table S5. Correlations Between Diversity Ideologies and Dependent Variables** | | | | | | | |  |  |
| --- | --- | --- | --- | --- | --- | --- | --- | --- | --- |
|  |  |  |  |  |  |  |  |  |  |
|  | *M* | *SD* | Value-in-group differences | Value-in-individual differences | Value-in-similarities | Equality | Value-in-diversity | Business Case | Moral Case |
| Support | 5.38 | .43 | .15 | <.01 | -.15 | .05 | .11 | -.13 | .09 |
| Tokenism | 4.74 | .46 | .31** | .13 | -.11 | .01 | .22* | -.15 | .11 |
| Word Count | 114.59 | 67.52 | .24* | .15 | .19 | .19 | .20* | .02 | -.04 |
| *Note.* * *p* ≤ .05 ** *p* ≤ .01 *** *p* ≤ .001 |  |  |  |  |  |  |  |  |  |

| **Table S6. Relationship Between Diversity Statement Components and Psychological Measures with Value-in-Group Differences and Moral Case in Model** | | | | | | | |
| --- | --- | --- | --- | --- | --- | --- | --- |
|  | **Support** | | |  | **Tokenism** | | |
|  | *R²* = .05, *F*_6,93_ = 0.80, *p* = .575 | | |  | *R²* = .12, *F*_6,93_ = 2.04 *p* = .068 | | |
| **Predictor** | **β** | ***t*** | ***p*** |  | **β** | ***t*** | ***p*** |
| Value-in-group differences | .14 | 1.29 | .202 |  | .28 | 2.62 | .010 |
| Value-in-individual differences | -.01 | -.12 | .905 |  | .07 | .70 | .485 |
| Value-in-similarities | -.12 | -1.08 | .284 |  | -.08 | -.72 | .474 |
| Equality | .03 | .30 | .763 |  | -.01 | -.05 | .958 |
| Moral Case | .09 | .85 | .397 |  | .11 | 1.09 | .277 |
| Word Count | -.03 | -.26 | .797 |  | .01 | .05 | .960 |

**Discussion**

We found that minoritised racial groups supported the moralistic value-in-diversity cluster more than the instrumental value-in-diversity cluster, although the overall test was not statistically significant. These clusters only differed in the presence of the moral case in the former and the business case in the latter, suggesting minoritised racial groups prefer the moral case. This benefit of the moral case over the business case is in line with previous research (Starck et al., 2021; Trawalter et al., 2016). The individual component analyses showed that for people from racial minority backgrounds, statements that mentioned value-in-group differences were associated with increased tokensim. Although unexpected, it dovetails somewhat with past research (Kirby & Kaiser, 2021) suggesting that a focus on value-in-group differences can make minoritised groups feel pressure to represent their racial group. We also found no significant relationships between the components and support from minoritised racial groups. The key limitations of this research are that it was underpowered and used a single item measure for tokenism. In the next stages of this research, we assessed whether the same patterns were observed in companies with a better powered study.

**Additional Tables**

**Table S7**

Relationship Between Diversity Statement Components and Psychological Measures with Value-In-Diversity and Moral Case in Model

|  | **Support** | | |  | **Tokenism** | | |
| --- | --- | --- | --- | --- | --- | --- | --- |
|  | *R²* = .04, *F*_6,93_ = 0.66, *p* = .682 | | |  | *R²* = .08, *F*_6,93_ = 1.33, *p* = .251 | | |
|  |  |  |  |  |  |  |  |
| **Predictor** | **β** | ***t*** | ***p*** |  | **β** | ***t*** | ***p*** |
| Value-in-diversity | .10 | .93 | .356 |  | .18 | 1.68 | .097 |
| Value-in-individual differences | -.01 | -.05 | .962 |  | .09 | .88 | .383 |
| Value-in-similarities | -.12 | -1.13 | .260 |  | -.09 | -.88 | .381 |
| Value-in-equality | .03 | .31 | .758 |  | -.01 | -.05 | .963 |
| Moral Case | .10 | .96 | .339 |  | .13 | 1.29 | .199 |
| Word Count | -.02 | -.14 | .890 |  | .04 | .33 | .743 |

**Table S8**

Relationship Between Diversity Statement Components and Psychological Measures with Value-In-Group Differences and Business Case in Model

|  | **Support** | | |  | **Tokenism** | | |
| --- | --- | --- | --- | --- | --- | --- | --- |
|  | *R²* = .05, *F*_6,93_ = 0.76, *p* = .605 | | |  | *R²* = .12, *F*_6,93_ = 2.01, *p* = .072 | | |
|  |  |  |  |  |  |  |  |
| **Predictor** | **β** | ***t*** | ***p*** |  | **β** | ***t*** | ***p*** |
| Value-in-group differences | .13 | 1.15 | .255 |  | .26 | 2.39 | .019 |
| Value-in-individual differences | <.01 | <.01 | >.999 |  | .09 | .86 | .394 |
| Value-in-similarities | -.10 | -.91 | .363 |  | -.05 | -.50 | .618 |
| Value-in-equality | .04 | .34 | .738 |  | <.01 | -.02 | .985 |
| Business case | -.08 | -.71 | .480 |  | -.11 | -1.02 | .311 |
| Word Count | -.03 | -.30 | .764 |  | <.01 | <.01 | .997 |

**Table S9**

Relationship Between Diversity Statement Components and Psychological Measures with Value-In-Diversity and Business Case in Model

|  | **Support** | | |  | **Tokenism** | | |
| --- | --- | --- | --- | --- | --- | --- | --- |
|  | *R²* = .04, *F*_6,93_ = 0.65, *p* = .691 | | |  | *R²* = .08, *F*_6,93_ = 1.41, *p* = .218 | | |
|  |  |  |  |  |  |  |  |
| **Predictor** | **β** | ***t*** | ***p*** |  | **β** | ***t*** | ***p*** |
| Value-in-diversity | .09 | .83 | .410 |  | .17 | 1.53 | .129 |
| Value-in-individual differences | .01 | .10 | .918 |  | .12 | 1.11 | .271 |
| Value-in-similarities | -.10 | -.92 | .359 |  | -.06 | -.57 | .570 |
| Value-in-equality | .04 | .34 | .737 |  | <.01 | -.02 | .981 |
| Business case | -.10 | -.93 | .355 |  | -.15 | -1.46 | .147 |
| Word Count | -.02 | -.20 | .840 |  | .03 | .24 | .811 |

**Study 2**

**Additional Tables**

| **Table S10**  *Levels of Value Fit, Tokenism, Authenticity, Company Interest by Cluster* | | | | | | | | |
| --- | --- | --- | --- | --- | --- | --- | --- | --- |
|  | **Value Fit** | | **Tokenism** | | **Authenticity** | | **Company Interest** | |
| Cluster | *M* | *SD* | *M* | *SD* | *M* | *SD* | *M* | *SD* |
| 1. Instrumental Individualism | 5.12 ^a^ | .47 | 3.54 | .42 | 4.97 ^a^ | .55 | 5.11 ^a^ | .51 |
| 2. Moralistic Individualism | 5.12 ^a^ | .59 | 3.49 | .44 | 5.00 | .60 | 5.07 ^a^ | .61 |
| 3. Instrumental Value-In-Diversity | 5.43 ^b^ | .43 | 3.35 | .45 | 5.29 ^b^ | .50 | 5.43 ^b^ | .49 |

^a,b^ differing superscripts denote significant differences in mean levels of a dependent variable by cluster at *p* ≤ .05; specifically indicating that diversity statements conveying *Instrumental Value-in-Diversity* elicited a greater sense of value fit and company interest among respondents.

**Table S11**

Relationship Between Diversity Statement Components and Psychological Measures with Value-In-Diversity and Moral Case in Model

|  | **Interest** | | |  | **Value Fit** | | |  | **Authenticity** | | |  | **Tokenism** | | |
| --- | --- | --- | --- | --- | --- | --- | --- | --- | --- | --- | --- | --- | --- | --- | --- |
|  | *R²* = .23, *F*_6,241_ = 11.68, *p* < .001 | | |  | *R²* = .20, *F*_6,241_ = 9.74, *p* < .001 | | |  | *R²* = .17, *F*_6,241_ = 8.17, *p* < .001 | | |  | *R²* = .14, *F*_6,241_ = 6.63, *p* < .001 | | |
|  |  |  |  |  |  |  |  |  |  |  |  |  |  |  |  |
| **Predictor** | **β** | ***t*** | ***p*** |  | **β** | ***t*** | ***p*** |  | **β** | ***t*** | ***p*** |  | **β** | ***t*** | ***p*** |
| Value-in-diversity | .11 | 1.70 | .090 |  | .11 | 1.71 | .088 |  | .08 | 1.31 | .193 |  | -.05 | -.75 | .456 |
| Value-in-individual differences | .15 | 2.58 | .010 |  | .13 | 2.21 | .028 |  | .10 | 1.55 | .124 |  | -.06 | -.93 | .353 |
| Value-in-similarities | <.01 | -.03 | .975 |  | -.01 | -.22 | .823 |  | -.04 | -.70 | .487 |  | .03 | .53 | .600 |
| Value-in-equality | .07 | 1.12 | .265 |  | .09 | 1.32 | .189 |  | .06 | .87 | .387 |  | -.07 | -1.02 | .309 |
| Moral Case | <.01 | -.03 | .974 |  | <.01 | -.06 | .951 |  | .02 | .35 | .728 |  | -.05 | -.73 | .464 |
| Word Count | .33 | 4.92 | <.001 |  | .30 | 4.31 | <.001 |  | .31 | 4.39 | <.001 |  | -.30 | -4.16 | <.001 |

**Table S12**

Relationship Between Diversity Statement Components and Psychological Measures with Value-In-Group Differences and Business Case in Model

|  | **Interest** | | |  | **Value Fit** | | |  | **Authenticity** | | |  | **Tokenism** | | |
| --- | --- | --- | --- | --- | --- | --- | --- | --- | --- | --- | --- | --- | --- | --- | --- |
|  | *R²* = .23, *F*_6,241_ = 11.84, *p* < .001 | | |  | *R²* = .21, *F*_6,241_ = 10.48, *p* < .001 | | |  | *R²* = .18, *F*_6,241_ = 8.49, *p* < .001 | | |  | *R²* = .14, *F*_6,241_ = 6.55, *p* < .001 | | |
|  |  |  |  |  |  |  |  |  |  |  |  |  |  | | |
| **Predictor** | **β** | ***t*** | ***p*** |  | **β** | ***t*** | ***p*** |  | **β** | ***t*** | ***p*** |  | **β** | ***t*** | ***p*** |
| Value-in-group differences | .10 | 1.67 | .097 |  | .14 | 2.29 | .023 |  | .11 | 1.78 | .077 |  | -.05 | -.78 | .434 |
| Value-in-individual differences | .14 | 2.30 | .022 |  | .11 | 1.81 | .072 |  | .08 | 1.22 | .223 |  | -.05 | -.72 | .471 |
| Value-in-similarities | <.01 | -.04 | .967 |  | -.01 | -.17 | .863 |  | -.04 | -.65 | .516 |  | .03 | .54 | .591 |
| Value-in-equality | .07 | 1.09 | .279 |  | .08 | 1.32 | .189 |  | .06 | .89 | .377 |  | -.07 | -1.09 | .277 |
| Business case | .06 | .96 | .338 |  | .07 | 1.18 | .239 |  | .04 | .58 | .561 |  | -.01 | -.20 | .842 |
| Word Count | .34 | 5.27 | <.001 |  | .30 | 4.51 | <.001 |  | .31 | 4.64 | <.001 |  | -.31 | -4.45 | <.001 |

**Table S13**

Relationship Between Diversity Statement Components and Psychological Measures with Value-In-Diversity and Business Case in Model

|  | **Interest** | | |  | **Value Fit** | | |  | **Authenticity** | | |  | **Tokenism** | | |
| --- | --- | --- | --- | --- | --- | --- | --- | --- | --- | --- | --- | --- | --- | --- | --- |
|  | *R²* = .23, *F*_6,241_ = 11.95, *p* < .001 | | |  | *R²* = .20, *F*_6,241_ = 10.11, *p* < .001 | | |  | *R²* = .17, *F*_6,241_ = 8.24, *p* < .001 | | |  | *R²* = .14, *F*_6,241_ = 6.54, *p* < .001 | | |
|  |  |  |  |  |  |  |  |  |  |  |  |  |  |  |  |
| **Predictor** | **β** | ***t*** | ***p*** |  | **β** | ***t*** | ***p*** |  | **β** | ***t*** | ***p*** |  | **β** | ***t*** | ***p*** |
| Value-in-diversity | .11 | 1.82 | .070 |  | .12 | 1.85 | .066 |  | .09 | 1.38 | .169 |  | -.05 | -.78 | .435 |
| Value-in-individual differences | .14 | 2.36 | .019 |  | .12 | 1.96 | .051 |  | .08 | 1.35 | .179 |  | -.05 | -.76 | .449 |
| Value-in-similarities | <.01 | -.08 | .938 |  | -.02 | -.28 | .781 |  | -.04 | -.74 | .460 |  | .03 | .56 | .574 |
| Value-in-equality | .08 | 1.23 | .220 |  | .09 | 1.45 | .149 |  | .06 | .99 | .326 |  | -.08 | -1.15 | .253 |
| Business case | .07 | 1.12 | .264 |  | .08 | 1.33 | .186 |  | .04 | .69 | .489 |  | -.02 | -.27 | .789 |
| Word Count | .32 | 4.66 | <.001 |  | .28 | 4.02 | <.001 |  | .30 | 4.22 | <.001 |  | -.30 | -4.09 | <.001 |

References

Alin, A. (2010). Multicollinearity. *WIREs Computational Statistics*, *2*(3), 370–374. https://doi.org/10.1002/wics.84

Apfelbaum, E. P., Stephens, N. M., & Reagans, R. (2016). *Beyond One-Size-Fits-All: Tailoring Diversity Approaches to the Representation of Social Groups*. *111*(4), 547–566. https://doi.org/10.1037/pspi0000071

Gündemir, S., Dovidio, J. F., & Homan, A. C. (2017a). *The Impact of Organizational Diversity Policies on Minority Employees’ Leadership Self-Perceptions and Goals*. *24*(2), 172–188. https://doi.org/10.1177/1548051816662615

Gündemir, S., Homan, A. C., Usova, A., & Galinsky, A. D. (2017b). Multicultural meritocracy: The synergistic benefits of valuing diversity and merit. *Journal of Experimental Social Psychology*, *73*, 34–41. https://doi.org/10.1016/j.jesp.2017.06.002

Jansen, W. S., Kröger, C., Van der Toorn, J., & Ellemers, N. (2021). The right thing to do or the smart thing to do? How communicating moral or business motives for diversity affects the employment image of Dutch public and private sector organizations. *Journal of Applied Social Psychology*, *51*(7), 746–759. https://doi.org/10.1111/jasp.12783

Kirby, T. A., & Kaiser, C. R. (2021). Person-Message Fit: Racial Identification Moderates the Benefits of Multicultural and Colorblind Diversity Approaches. *Personality and Social Psychology Bulletin*, *47*(6), 873–890. https://doi.org/10.1177/0146167220948707

Plaut, V. C., Thomas, K. M., & Goren, M. J. (2009). Is Multiculturalism or Color Blindness Better for Minorities? *Psychological Science*, *20*(4), 444–446. https://doi.org/10.1111/j.1467-9280.2009.02318.x

Purdie-Vaughns, V., Steele, C. M., Davies, P. G., Ditlmann, R., & Crosby, J. R. (2008). Social Identity Contingencies: How Diversity Cues Signal Threat or Safety for African Americans in Mainstream Institutions. *Journal of Personality and Social Psychology*, *94*(4), 615–630. https://doi.org/10.1037/0022-3514.94.4.615

Starck, J. G., Sinclair, S., & Shelton, J. N. (2021). How university diversity rationales inform student preferences and outcomes. *Proceedings of the National Academy of Sciences*, *118*(16), e2013833118. https://doi.org/10.1073/pnas.2013833118

Trawalter, S., Driskell, S., & Davidson, M. N. (2016). What Is Good Isn’t Always Fair: On the Unintended Effects of Framing Diversity as Good: On the Unintended Effects of Framing Diversity as Good. *Analyses of Social Issues and Public Policy*, *16*(1), 69–99. https://doi.org/10.1111/asap.12103

Wolsko, C., Park, B., Judd, C. M., & Wittenbrink, B. (2000). Framing interethnic ideology: Effects of multicultural and color-blind perspectives on judgments of groups and individuals. *Journal of Personality and Social Psychology*, *78*(4), 635–654. https://doi.org/10.1037/0022-3514.78.4.635

1. For White participants, support also did not differ across clusters (*F*(4,90) = 1.27, *p* = .288, η_p_^2^ = .05) and no differences between the clusters were found in the subsequent post-hoc comparisons. [↑](#footnote-ref-1)
2. For White participants, tokenism also did not differ across clusters (*F*(4,90) = 1.44, *p* = .228, η_p_^2^ = .06), however, the post-hoc analyses revealed that White people feel more tokenised by the moralistic value-in-diversity, dual identity, and moral equality clusters than the instrumental equality cluster. [↑](#footnote-ref-2)
3. We found that statements that focused on value-in-individual differences (*r*(100) = .21, *p* = .032) or value-in-diversity (*r*(100) = .24, *p* = .016) were associated with more support by White people in the correlations but these effects did not hold in the regressions. [↑](#footnote-ref-3)
